# A Tale of Two Viruses: The Distinct Spike Glycoproteins of Feline Coronaviruses

**DOI:** 10.3390/v12010083

**Published:** 2020-01-10

**Authors:** Javier A. Jaimes, Jean K. Millet, Alison E. Stout, Nicole M. André, Gary R. Whittaker

**Affiliations:** 1 Department of Microbiology & Immunology, College of Veterinary Medicine, Cornell University, Ithaca, NY 14853, USA; jaj246@cornell.edu (J.A.J.); aek68@cornell.edu (A.E.S.); nma39@cornell.edu (N.M.A.); 2 Virologie et Immunologie Moléculaires, INRAE, Université Paris-Saclay, 78352 Jouy-en-Josas, France; jean.millet@inra.fr

**Keywords:** feline coronavirus, feline infectious peritonitis, spike protein, serotype, genetic characterization

## Abstract

Feline coronavirus (FCoV) is a complex viral agent that causes a variety of clinical manifestations in cats, commonly known as feline infectious peritonitis (FIP). It is recognized that FCoV can occur in two different serotypes. However, differences in the S protein are much more than serological or antigenic variants, resulting in the effective presence of two distinct viruses. Here, we review the distinct differences in the S proteins of these viruses, which are likely to translate into distinct biological outcomes. We introduce a new concept related to the non-taxonomical classification and differentiation among FCoVs by analyzing and comparing the genetic, structural, and functional characteristics of FCoV and the FCoV S protein among the two serotypes and FCoV biotypes. Based on our analysis, we suggest that our understanding of FIP needs to consider whether the presence of these two distinct viruses has implications in clinical settings.

## 1. Introduction

The Coronaviridae family comprises a diverse group of viruses that affects birds and mammals (including humans), resulting in a variety of disease manifestations spanning respiratory, gastrointestinal, neurological and other tissue tropisms [1]. The diversity of viruses is encompassed by four viral genera, namely the alpha-, beta-, gamma-, and delta-coronaviruses. Among the coronaviruses (CoVs), severe acute respiratory syndrome coronavirus (SARS-CoV) and the Middle East respiratory syndrome coronavirus (MERS-CoV), both betacoronaviruses, have received special attention as emergent pathogens in humans, with the potential to create global epidemics [2,3,4]. However, CoVs are also well known as important pathogens in both domesticated and wild birds and mammals [5]. Felids are no exception to this, and feline coronavirus (FCoV) is known to be the cause of disease in both wild and domestic cats [6,7,8]. FCoV is grouped as a member of the *Alphacoronavirus* genus, along with a range of other coronaviruses causing disease in dogs, pigs, and humans, as well as other mammalian species.

FCoV has been a focus of study for several decades due to its interesting behavior in the infected animal and its ability to cause the systemic, often lethal illness, feline infectious peritonitis (FIP). The virus is commonly proposed to exist as two forms or biotypes, either causing mainly sub-clinical disease (where it is known as feline enteric coronavirus, or FECV) or an aggressive and severe form, where it is known as feline infectious peritonitis virus (FIPV) [9]. While there is still no definitive evidence to understand the transition between the two forms, several authors have reported that mutations in the FCoV genome result in changes to the pathogenicity and tropism of the virus, with a significant role for infected macrophages resulting in a severe-systemic and frequently lethal disease in cats [10,11,12,13,14]. However, the dichotomous behavior (i.e., FECV-FIPV) has been questioned more recently with evidence of “systemic” FCoVs that are not “FECVs” by definition, but that do not cause FIP either (therefore cannot be considered as FIPVs), contributing to the diversity of FCoV and refuting the discrete FECV-FIPV concept. [13]. While remaining a devastating disease in cat populations, recent reports describing the role of nucleoside analogs and protease inhibitors, and their potential as a treatment for FCoV infection have raised the possibility of preventing lethal FIP [15,16,17,18].

Conventionally, FCoVs have been regarded to exist as two distinct serotypes, based on significant antigenic differences between different viruses infecting cats. The designation of two different serotypes initially based on antigenic differences found through characterization of spike-specific monoclonal antibodies (MAbs) against FCoV and canine coronavirus (CCoV) strains [19,20]. From these studies, FCoVs corresponding to each serotype have been described: feline (serotype I or type I) and canine (serotype II or type II) [21,22]. While both serotypes have been categorized in FECV and FIPV forms, both of which can cause FIP, serotype I viruses are much more prevalent in cat populations and so are the leading cause of FIP [23,24,25,26]. Such viruses grow only poorly in cell culture and are hence understudied compared to serotype II viruses, which have arisen by independent recombination events with canine coronaviruses and replicate well in cell culture [27,28].

According to established virus taxonomical criteria, all FCoVs are grouped as single species (*Alphacoronavirus 1*), along with canine coronaviruses and transmissible gastroenteritis virus (TGEV) of swine, a grouping based on sequences of whole genomes or the conserved ORF1ab gene. It is clear, however, that this single species designation does not reflect the significant differences between these viruses. In an attempt to provide a more refined and biologically-relevant grouping, we recently provided evidence for such classification based on the viral spike protein, which is a major driver of viral tropism and pathogenesis [29]. The study showed that the *Alphacoronavirus 1* species can be sub-grouped such that the different FCoVs are present in distinct clades, which classically would be defined as monophyletic groups (as they share a common ancestor) but with members of each clade sharing a set of distinctive characteristics [29]. In this designation, serotype I and serotype II FCoVs have sufficiently distinguishing features to define them as separate biological entities, and so could be considered to be distinct virus types.

Here, we address new concepts related to the non-taxonomical classification and differentiation among FCoVs. To do this, we analyzed and compared the genetic, structural and functional characteristics of FCoV and the FCoV S protein among the two serotypes and the two biotypes and conclude that serotype I and serotype II FCoVs are highly distinct viruses, which we will refer to as type I and type II. We suggest that our understanding of FIP should consider the presence of these two distinct viruses, in order to determine whether or not it has implications in clinical settings. Such distinctions are suggested based on documented differences seen in cell culture between type I and type II viruses, for example with regard to interferon responses and the activity of antiviral drugs [30,31].

## 2. Feline Coronaviruses as Agents of Disease

FCoV, like all coronaviruses, is an enveloped virus with a large (~30 kb) single-strand positive-sense RNA genome (ssRNA+) [1,32]. The FCoV genome encodes 11 proteins with four structural proteins, namely spike (S), envelope (E), matrix (M), and nucleocapsid (N), and five non-structural proteins, namely the replicase 1a and 1b polyproteins (which are enzymatically cleaved to produce 16 functional proteins involved in RNA synthesis), and the accessory proteins 3a, 3b, 3c, 7a, and 7b [6,33]. While all viral proteins play a role during FCoV infection and replication, the structural proteins have the important role of protecting the viral genome and facilitating the interactions of virions with susceptible cells. The N protein binds to the viral genomic RNA to protect it, while the M and E proteins interact with the cell membrane and play a role during the maturation and assembly of the virus [6]. The E protein has been also reported to play a major role in the regulation of pathogenicity of other CoVs [34]. The S protein is known to be viral regulator of the cell entry and the major antigenic element of the virus [35,36,37]. Several studies have implicated this protein as the main driver of changes in viral tropism and virulence [11,12,13,38]. In addition, the historical serotype classification of FCoV S, based on antigenic differences, reaffirms the importance of this protein in FCoV pathogenesis [21,22].

The extensive available literature has described that the initial FCoV infection is localized to the enteric tract, with this infection linked to the “FECV” biotype and typically being subclinical or causing only mild enteric disease [6]. However, in some cats, this initial infection can lead to extra-enteric infections in other organs [6,8], and after initial infection, the virus is believed to mutate into the “FIPV” biotype and produce a severe and often lethal disease called FIP, if left untreated [39,40]. Both biotypes have been described for the two FCoV serotypes. The commonly accepted model is that, in 5–10% of the cases, primary infection (by “FECV”) progresses to a systemic form that is characterized by an acute accumulation of liquid in the peritoneal and thoracic cavities (known as the “wet” or “effusive” form), hence the name feline infectious peritonitis [41,42]. In some cases, the host’s immune response can alter the clinical manifestations of the FCoV infection, leading to a less aggressive, but equally relevant FIP form, where the accumulation of fluid is not evident (the “dry” or “non-effusive” form) and other major clinical signs arise (e.g., neurological signs and uveitis) [43,44]. This concept has been recognized as the sole model of FCoV pathogenesis, and while it describes key elements in this disease, it may fall short in describing the complexity and diversity of FCoV infection clinical outcomes.

## 3. Genetic Characterization of FCoV

**Figure 1:**
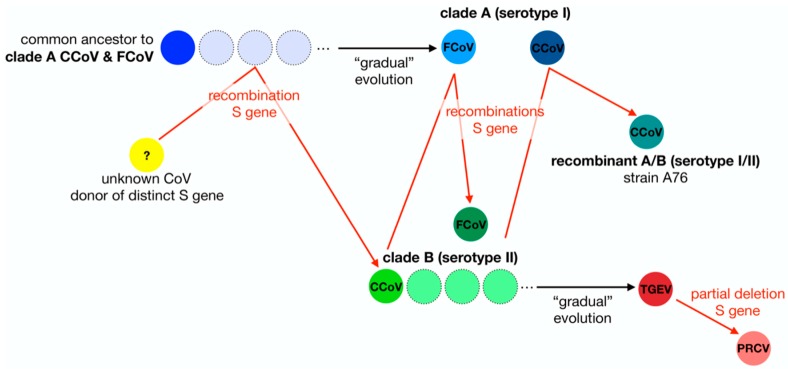
Diagram of the S gene flow within the Alphacoronavirus 1 species. In this schematic diagram the evolution of members of the *Alphacoronavirus 1* species is summarized using a S gene-centric view. It is thought that clade A (serotype I) CCoV and FCoV share a common ancestor. A recombination event with an unknown coronavirus allowed the emergence of a clade B (serotype II) CCoV with a S protein distinct from clade A CCoV. Multiple independent recombination events led to the emergence of clade B (serotype II) FCoV and recombinant A/B (serotype I/II) CCoV. TGEV is believed to have emerged from clade B CCoV. Partial deletion of the N-terminal domain of the S protein of TGEV gave rise to PRCV.

To gain a better understanding of the distinctive features of the two serotypes of FCoV, it is important to consider the origins and evolutionary history of FCoV in the context of the other members of the *Alphacoronavirus 1* species (Figure 1). The evolution of FCoV and CCoV (like that of many RNA viruses) is complex, but it is believed they originate from a common ancestor. During their evolutionary process, it has been shown that several recombination events have led to the emergence of CCoVs and FCoVs in their respective animal populations [28,29,45]. In the case of FCoV, recombination events between FCoV and CCoV genomes resulted in the appearance of novel, chimeric FCoVs whose S proteins have their origin in CCoV [28]. As a result of these recombination events, FCoV type I and II differ both genetically and antigenically.

Early FCoV studies have shown that CCoV antibodies are able to bind to type II FCoV, but fail to bind type I FCoV [20,22]. This denotes major differences in antigenicity of the viruses, and also reinforces findings that the biology of the two viral types (in particular with regard to receptor usage and cell culture adaptation) differ greatly with type II FCoV being the most easily isolated and grown in cell culture [46,47]. More recent work by Terada and colleagues analyzed the dynamics of recombination events between FCoV and CCoV [48]. In their research, the authors compared the sequences of type II FCoV and type II CCoV, to study the mechanisms of emergence of type II FCoVs, as well as to determine potential recombination sites. Their results showed that, while the three studied FCoV type II strains emerged from homologous recombination between type I FCoV and type II CCoV viruses, they possessed different recombination sites, suggesting that the mechanisms of emergence of these viruses are independent and unique to each strain, rather than uniform for all FCoV type II. Interestingly, in all the strains, the S protein shared the same characteristics at the S1/S2 interface, which we will discuss later. This contrasts with another study where the horizontal transmission of identical FCoV type II viruses was reported [49]. In this report, an outbreak in an animal shelter was studied and FCoV type II viruses were consistently detected in all the deceased animals, suggesting the effective transmission of these viruses among the cats in this outbreak. Additionally, type I viruses were also detected in other animals in the same facility, indicating the co-circulation of the two viruses and rising questions about the possibility of FCoV type I and II coinfections and the possible consequences for the host. While these two studies give different views of type II FCoV, it is important to note that the typing method used by Wang et al. focused on the analysis of the 3′ end of spike and the 3a and 3c genes rather than full genome analysis as described by Terada and collaborators, rendering the side to side analysis of these two studies inconclusive.

A recent study from our group suggested the inclusion of new parameters for the classification within the *Alphacoronavirus 1* species, which includes FCoV as one of its representative members [29]. In this study, we highlighted that the current taxonomic classification scheme (based on key domains of the CoV replicase polyprotein (ORF1ab) and devised by the International Committee on Taxonomy of Viruses (ICTV)), ignores genetic differences in the S gene that are key in differentiating FCoV types. Analyses based on the S1 and S2 domains, and on the full-length S gene of *Alphacoronavirus 1*, found that the viruses branched as two distinct clades where type I FCoV viruses grouped with type I CCoV and recombinants (clade A—type I) and type II FCoV viruses grouped with CCoV II and TGEV viruses. This S-centric phylogenetic grouping aligned well with the historical serotype classification of both FCoV and CCoV, and when other alphacoronaviruses were taken into account, the grouping also matched well with current taxonomical classification, suggesting that S gene-based phylogenetic analyses could provide more accurate and meaningful sub-species classification.

The results of the phylogenetic analysis offer new insights on FCoV classification, as well as provide more evidence for the major differences between the two types. One of the main functional differences of the FCoV S protein between the two types or clades is their proteolytic processing requirements [12,50]. It has been described that the S protein of FCoV type I viruses possess two distinct activation sites: the S1/S2 site which is located at the interface between the S1 and the S2 domains, and the S2′ site which is located immediately upstream of the fusion peptide (FP) [29,51]. In contrast, the S protein of FCoV II viruses are believed to possess a single activation site at S2′, a characteristic that is also shared with the majority of other alphacoronaviruses [2]. The additional cleavage site of the FCoV type I spike protein, introduces additional steps in its activation pathway that suggest differences in molecular mechanisms of its function. The S1/S2 site has been described for the S proteins of other CoV mostly belonging to the *Betacoronavirus* and *Gammacoronavirus* species. However, phylogenetic evidence did not show the homology of the S gene between FCoV type I and that of betacoronaviruses [29].

Taking an S gene-centric view, the evolutionary histories of FCoV and other members of the *Alphacoronavirus 1* species depicted in Figure 1 highlights the many recombination events of the S gene as well as the non-linear aspect of the evolution of members of *Alphacoronavirus 1* species. The frequent occurrence of recombination events and deletions is precisely why reconstructing virus phylogenies is often difficult. Interestingly, in the case of *Alphacoronavirus 1* there is a precedent for viruses that depart from classical linear evolution, because of an important modification in the S gene. Indeed, a deletion in the N-terminal part of the S protein of TGEV, which replicates in intestinal enterocytes and is responsible for severe diarrhea in young pigs, gave rise to the emergence of a different virus named porcine respiratory coronavirus (PRCV), which has an altered tissue tropism and infects respiratory tract epithelial cells causing mild to subclinical infections [52].

From the diagram in Figure 1, it is clear to see why previous classification schemes based solely on phylogenetic analyses that do not take into account the flow of S gene exchanges and modifications (deletions) would fail to capture the complex relationships, histories and distinctive characteristics of this group of coronaviruses. We believe that, much like the situation with TGEV and PRCV, and despite the fact that type I (clade A) and type II (clade B) FCoV are grouped within the same species, the distinct origins of their S genes and the significant functional differences between them is such that they can be considered as two separate biological entities.

## 4. Growth Properties of FCoV in Cell Culture

FCoV type II viruses have uncertain and/or complex origins, but have been shown to be readily isolated and grown in cell culture. This has facilitated their study and allowed the identification of the type II cellular receptor, aminopeptidase N (APN), and the propagation of laboratory-adapted strains, such as the well-studied FCoV-WSU-79-1683 (FECV II 1683) and FCoV-WSU-79-1146 (FIPV II 1146) strains [28]. It is important to mention that while the FCoV-WSU-79-1683 strain has been considered an FECV (hence the name FECV II 1683), there is strong evidence to consider that this strain did not behave as an FECV when it was originally isolated [53,54,55]. In contrast, our knowledge of the molecular mechanisms involved in type I virus infections is limited to a highly restricted number of cell culture-adapted viruses such as FIPV-Black (TN406), which remains often difficult to grow in culture [22]. As a result of this, the vast majority of in vitro, ex vivo, and in vivo FCoV studies have been carried out using type II strains which are epidemiologically less common and do not represent well the circulating population of type I viruses. This is a major limitation in our understanding of FIP. While most of the available studies conducted with type II viruses are often accepted as comparable with type I FCoV viruses, there is sufficient genetic, structural and functional evidence to support that the S protein of type II FCoV (which regulates most of the viral entry processes) is significantly different to its homologue in FCoV I strains, suggesting that the pathogenesis of the two types may also differ.

## 5. The Coronavirus S Protein

**Figure 2:**
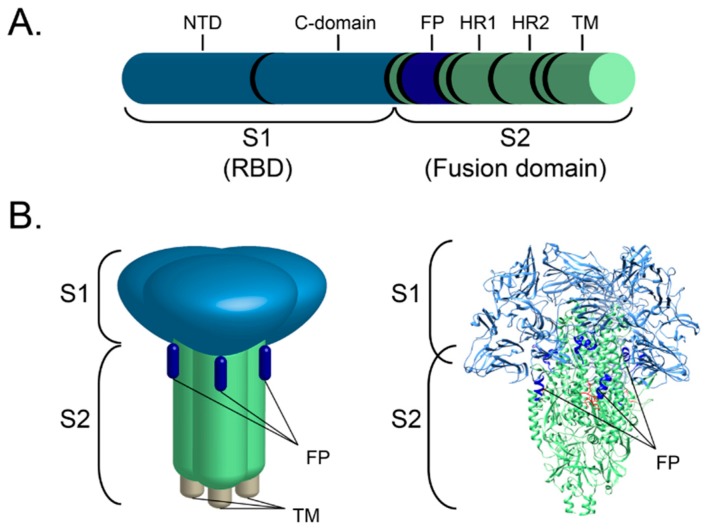
The FCoV S gene and protein functional components. A schematic of the FCoV S gene (**A**) and the S protein (**B** left), the S1 domain or RBD forms the head of the protein, while the S2 domain (fusion domain) forms the stalk of the protein. Trimeric structure of the S protein of HCoV-NL63 (PDB # 5SZS) (**B** right). The structure of the transmembrane domain has not yet been solved. The two domains S1 (blue) and S2 (green), as well as the major functional components RBD (NTD and C-domain), FP (bright blue), HR1, HR2 and the transmembrane domain (TM) are noted.

The coronavirus S protein is a large (approximately 200 kDa) class I viral fusion protein that is distributed around the viral envelope and is considered as the main viral regulator of entry into host cells [36,37,56]. As with other class I fusion proteins, S is mainly characterized by α-helix secondary structure [37,57]. The functional S protein is composed of three individual monomers organized in a trimeric form, and while there is no evidence regarding the number of FCoV S trimer units necessary to induce fusion, studies conducted on the prototypical class I influenza hemagglutinin (HA) have reported that at least three fusion protein trimers are necessary to induce viral-cell membrane fusion [36,58]. S is considered to be the major regulator of entry into the host cell [35]. To accomplish its function, the S protein undergoes a series of structural rearrangements that allow the exposure of an important functional segment of the fusion protein, called the fusion peptide (FP). The S protein is composed of three main domains: an ectodomain (which contains most of the functional elements to bind to the host cell receptor and induce fusion), a transmembrane domain, and a small endodomain (a short terminal tail or cytoplasmic domain) [56]. Within the ectodomain, two basic functional subunits have been described and named S1 and S2 (Figure 2).

The S1 subunit contains the receptor binding domain (RBD), the portion of the protein that interacts with the cellular receptor [59]. The S1 subunit forms the “head” of the protein (Figure 2) and can be further divided into two functional domains: a N terminal domain (NTD) and a C-terminal domain (C-domain). The NTD of several CoVs have been described to bind to carbohydrate receptors like 9-*O*-acetylated neuraminic acid in BCoV and human coronavirus HCoV-OC43, or α-2,3-linked sialic acid and heparan sulfate (HS) in IBV [60,61,62]. However, in contrast to the carbohydrate-binding examples, MHV NTD binds a cellular receptor, carcinoembryonic antigen-related cell adhesion molecule (CEACAM1) [63]. On the other hand, the C-domain is known to bind only protein receptors. Among these protein cellular receptors, angiotensin I converting enzyme 2 (ACE2) has been described as a receptor for HCoV-NL63 and SARS-CoV, dipeptidyl peptidase 4 (DPP4) for MERS-CoV, and aminopeptidase N (APN) has been described as suitable receptors for TGEV, PEDV and HCoV-229E, as well as for type II (clade B) FCoV [59,64,65].

The S2 domain includes the critical fusion peptide (FP), in addition to the two heptad repeats 1 and 2 (HR1 and HR2), the transmembrane domain and the terminal cytoplasmic tail (endodomain) (Figure 2) [56,59]. The FP is one of the most important components of the S2 domain. The FP is the primary regulator of virus-cell membrane fusion and its function is controlled by a series of molecular events, including structural changes due to protease activation, pH changes, and ion binding [66,67]. While there are several molecular and structural events necessary for the FP to be exposed and to fulfill its function, activation of the S protein by cellular proteases is perhaps the most critical of those events. As mentioned previously, protease activation can occur at different sites in fusion proteins, but CoV spikes are commonly activated at a site immediately upstream of the fusion peptide, which is called S2′. The other activation site, located at the interface between the S1 and the S2 domains and called S1/S2, is found in *Betacoronaviruses* and *Gammacoronaviruses*, but also in type I *Alphacoronavirus 1* FCoV (but not type II), suggesting a key difference in the molecular mechanisms used by the two types of this virus to gain entry into the cell [35].

## 6. Structural Differences between FCoV I and FCoV II S Protein

In the past few years, several cryo-electron microscopy structures of CoV S have been solved and reported, including the S proteins of SARS-CoV, MERS-CoV, MHV, HCoV-NL63, porcine deltacoronavirus (PDCoV) and IBV [68,69,70,71,72,73]. However, the structure of the FCoV S protein has not been solved yet, hindering the study of this protein and its structural properties. To address that, we initially used in silico analysis using structural models of three FCoV S, based on the structure of the HCoV-NL63 S [35]. Major structural differences between the studied FCoV type I and type II S proteins were predicted in the S1/S2 region, that include the specific S1/S2 cleavage site and a 10 amino acid loop flanking this site in FCoV I S from the strain FCoV-TN406 (also known as FIPV I Black), but missing in type II spikes from the strains FCoV-WSU-79-1683 and FCoV-WSU-79-1146. A similar finding performed at the amino acid sequence level was described in another report, showing that the S1/S2 cleavage site and the 10 amino acid insertion were also present in other FCoV I strains, as well as in the type I CCoV strain CCoV 23/03 [29]. These findings suggest major differences in the activation processes and the molecular pathways of the two FCoV serotypes, which can also affect the overall pathogenesis of each type of the virus.

**Figure 3:**
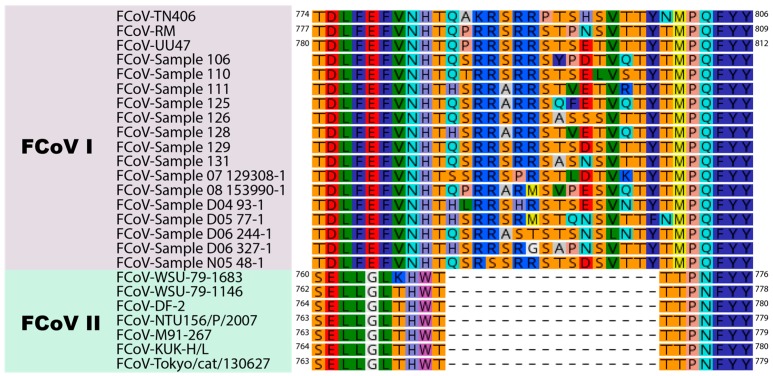
Alignment of the S1/S2 region of FCoV I and FCoV II strains. Sequences corresponding to the S1/S2 region of three FCoV type I strains (FCoV-RM, FCoV-UU47 and FCoV-TN406) and three FCoV type II strains (FCoV-DF-2, FCoV-WSU-79-1683 and FCoV-WSU-79-1146) reported in GenBank, and 15 FCoV type I sequences from clinical samples reported in the European Nucleotide Archive were aligned using the Geneious Prime 2019 (v.2019.2.3) software package (supplementary information 1). A 16 amino acid insertion including the S1/S2 cleavage site (6 amino acid) and a 10 amino acid flanking region was only observed in type I strains. FCoV type I sample are partial sequences, amino acid nomenclature cannot be displayed.

**Figure 4:**
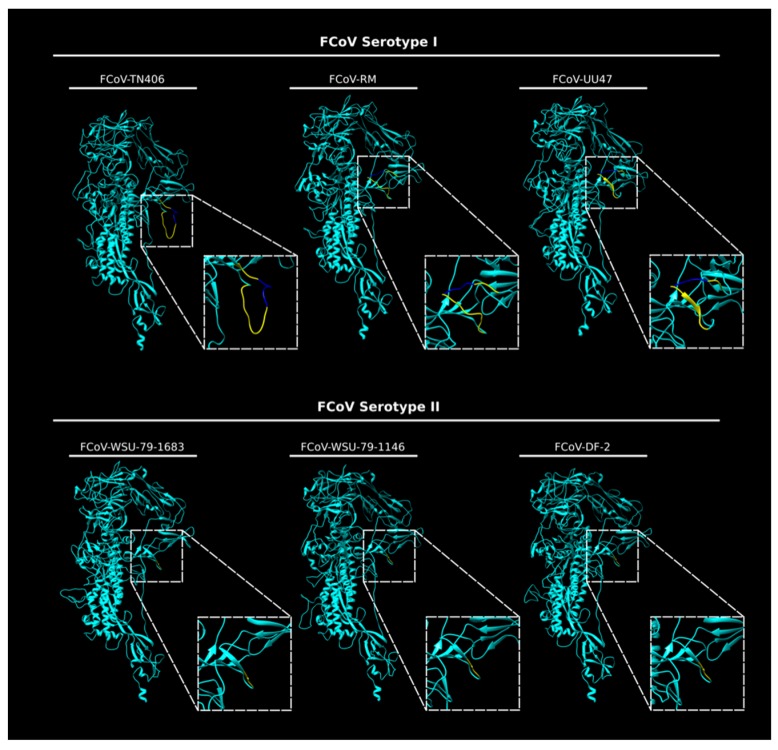
In silico modeling of the S protein from FCoV I and FCoV II strains. *In silico* FCoV S models of three FCoV type I strains (FCoV-RM, FCoV-UU47 and FCoV-TN406) and three FCoV type II strains (FCoV-DF-2, FCoV-WSU-79-1683 and FCoV-WSU-79-1146) were built using Chimera (UCSF Chimera v. 1.13.1, University of California). The S1/S2 region (dashed squares) was magnified to better visualize the differences between the two serotypes. The 16 amino acid insertion corresponding to the S1/S2 site (bright blue) and the 10 amino acid flanking region (yellow) were predicted as a flexible loop only in the FCoV type I S models.

Here, we extend our structural studies and compare the S protein sequence of three prototypical FCoV type I strains and three prototypical type II strains. Briefly, we compared the amino acid sequences corresponding to the interface region between the S1 and the S2 domain of eighteen type I and seven type II FCoV strains, through a pairwise alignment to determine differences between type I and type II FCoVs at the S1/S2 cleavage site and surrounding sequences. Indeed, we observed that the S1/S2 site and the 10 amino acid flanking sequence were only present in FCoV I strains (Figure 3). This 16 amino acid loop was present in the FCoV type I analyzed sequences detected clinically by our laboratory and also reported previously (Figure 3) [12]. In this analysis, we also included the following type II FCoV sequences: M91-267, KUK-H/L and Tokyo/cat/130627, reported by Terada et al. (2014) [48]. Interestingly, the three reported type II FCoV S sequences also shared the same characteristics at the S1/S2 interface, as they all lacked the S1/S2 cleavage site and the 10 amino acid flanking sequences (Figure 3). It was previously reported that these three strains resulted from recombination events between FCoV type I and CCoV type II strains, at different recombination sites, suggesting the FCoV type II strains resulted from independent recombination events [48]. However, the fact that these strains share the same characteristics at the S protein level highlights its importance in the FCoV S biology and suggest a key role in the biology of this type of virus. We also performed an in silico modeling of the S protein from six FCoV strains (three FCoV I and three FCoV II), following the methods described in [35]. Briefly, FCoV S protein models were predicted based on the structure of the HCoV-NL63 S protein (PDB ID: 5SZS). We first performed a pairwise alignment of amino acid sequences of the HCoV-NL63 S and each of the to-be-modeled FCoV S. Then, we used the Modeller tool (Modeller, v. 9.23, University of California) within the Chimera software package (v. 1.13.1, University of California) to build a distance-comparison model of each FCoV S protein. This methodology was previously described and used [35]. As observed previously, the S protein models showed differences at the S1/S2 region between the two FCoV serotypes. In the type I S models, a loop was observed corresponding to the S1/S2 cleavage site and the 10 amino acid insertion in the flanking regions of this site (Figure 4). The loop is predicted to be exposed in the S monomers, but differences in the folding and topology of the loop among the modeled S proteins suggest it can have some flexibility in the structure (Figure 4, compare between FCoV I S models). In contrast to the S1/S2 region of type I spike protein models, the serotype II S proteins lack the 16 amino acid insertion (including the S1/S2 site) and the models predicted a similar conformation at this region among the three studied proteins, suggesting that this site is not necessary for the FCoV II S function (Figure 4, compare the FCoV II S models). The absence of the S1/S2 cleavage site in FCoV serotype II is a major structural and functional difference when compared to serotype I FCoV S proteins, and its implications for the S protein functionality are yet to be addressed in the literature. However, these differences suggest that the mechanisms used by each serotype to induce fusion between the viral and the cell membrane could be significantly different.

## 7. Receptor Binding of FCoV S

Binding to a cellular receptor is the first step in the viral infection of a host cell, and in FCoV the S protein is the component carrying out this function. The cellular receptor used by FCoV has been a topic of discussion for several years. The primary receptor that has been described for FCoV is the feline aminopeptidase N (fAPN) with the possibility of additional binding to lectin molecules such as DC-SIGN, which would be considered a non-specific receptor, or attachment factor [74,75,76].

fAPN (also called CD13) is a membrane glycoprotein with metalloproteinase activity that is expressed in a variety of tissues, including: granulocytes, monocytes, fibroblasts, endothelial cells, synaptic membranes and several epithelial cells [64,77]. This receptor was first shown to be the key FCoV receptor by Tresnan et al. [64] in 1996, where it was shown to be a common receptor for many alphacoronaviruses, including the type I strain UCD-1. However, subsequent follow up studies have shown that this receptor appears to be suitable for FCoV type II, but not for type I [74,78]. As such, the primary receptor for serotype I viruses remains to be identified. While these major differences in receptor usage are likely an underlying reason behind the known differences in virus isolation between the type I and type II viruses in laboratory settings, other factors may also play a role, including availability of proteases.

## 8. Activation and Fusion of FCoV S

Following receptor binding, the subsequent step in virus entry is the fusion of the virus envelope with the host cell membrane. Fusion is mediated via the S2 domain and controlled by proteolytic cleavage events at one or two positions. The cleavage of type II FCoV occurs at a single position immediately adjacent to the viral fusion peptide (S2′), whereas cleavage of type I viruses occurs at both the S2′ site and at the interface between the S1 and S2 domains (the S1/S2 site). In the case of type I viruses, changes in viral tropism have been strongly linked to mutations in the S1/S2 site. However, mutations are variable between different cats and tissues [10,12]. The overall model is that the S1/S2 region consists of an exposed loop, highly susceptible to proteolytic cleavage and consisting of a recognition site for furin (a ubiquitous intracellular protease) and that mutations in the exposed loop lead to an elimination of furin cleavage, possibly accompanied by a switch to a distinct protease in distinct cell types and organs and transition to FIPV [12]. The finding of a distinct R793M mutation in the spike S1/S2 site in certain neurological samples of cats with FIP would be in line with this model [10]. How mutations in the S2′ site (shared between type I and type II viruses) link to pathogenesis is currently less clear. Early studies on S2′ site sequence changes and protease differences between the serotype II laboratory strains FIPV II 1146 and FECV II 1683 do not appear to be widely reproduced in clinical samples [79,80]. Studies on the S2′ site of serotype I viruses also do not show a clear linkage with disease outcome, although the site can often be mutated in viruses from FIP cases [80]. Overall however, these significant differences in cleavage-activation underlies the different structure-function relationships between type I and type II viruses and suggest a strong link to disease outcome.

For type I viruses, a mutation in a region within S2 (M1058A) also appears to correlate with disease outcome [13]. Mutation at this amino acid was first described by Chang et al. in 2012, were they described that this mutation allowed to distinguish FECV from FIP in most of their analyzed cases [11]. The region of S containing M1058 was originally attributed to a fusion peptide region, but is in fact down-stream of what is now considered the bona fide fusion peptide (based on the SARS-CoV fusion peptide) [66]. However, M1058 is adjacent to the HR1 domain and so may modulate fusion in some capacity. In a subsequent study, it was shown that the mutation M1058L was in fact indicative of systemic spread of FCoV, finding that a methionine (M) was present in fecal samples from both FIP and non-FIP cats, while leucine (L) was detected in tissues of both FIP and non-FIP cats, suggesting that this mutation cannot predict the potential of FCoV to cause FIP [13]. This mutation has only been studied in FCoVs corresponding to clinical cases that are most likely caused by type I viruses, so its role in type II FCoV is still unknown.

## 9. A Tale of Two Viruses

FCoV is a complex viral agent that causes a variety of clinical manifestations in cats. The virus has been the focus of study for several decades, but the available literature still fails to completely understand the nature and biology of this agent. Since the 1980s, it has been known that FCoV can occur in two different types based on antigenic and genetic differences in the S protein [21,22]. Here, we present evidence that the differences in the S protein are much more than serological or antigenic variants, resulting in the effective presence of two distinct viruses.

We believe that the current paradigm for FCoV does not fully take into consideration the two distinct viruses that circulate in cat populations. Our ability to molecularly diagnose FCoV infections in cats is limited, and while efforts are being made to discriminate between the different pathogenic forms of the virus (“FECV”, FIPV”, and “systemic”), information on the virus type (I or II) is not currently built into current clinical or molecular testing regimes. We believe the distinct differences in the S protein structure and function reviewed here are likely to translate into equally distinct biological and clinical outcomes. Evidence for such biological differences is already apparent in laboratory settings, based on the clear and well demonstrated differences in genetic background, structural features, receptor usage and protease activation between the type I and type II S proteins. Whether these differences translate into clinical settings remains opaque. However, the fact that molecular or antigenic differentiation between the two types are not usually considered in routine diagnostic testing, does not allow to understand the potential role of type II viruses in clinical settings. The realization that systemic forms of FCoV may exist beyond the FECV-FIPV paradigm should be more fully considered. Without consideration of the “type” of FCoV preset, our knowledge of FCoV transmission, pathogenesis, and clinical impact leaves unanswered questions.

## Supplementary Files

Supplementary File 1
