# Supplementary material for: A Tale of Two Viruses: The Distinct Spike Glycoproteins of Feline Coronaviruses
Source: Viruses. 2020 Jan 10;12(1):83. doi: 10.3390/v12010083 (PMC7019228; doi:10.3390/v12010083)
Supplement: Supplementary File 1 [file viruses-12-00083-s001.pdf]

Supplementary information 1

Sequence name	Type	Data base	Accession Number
FCoV-TN406	I	Not submitted	Courtesy of Susan Baker, Loyola University Chicago
FCoV-RM	I	NCBI GenBank	ACT10854.1
FCoV-UU47	I	NCBI GenBank	JN183882.1
FCoV-Sample 106	I	European Nucleotide Archive	HF954926.1
FCoV-Sample 110	I	European Nucleotide Archive	HF954927.1
FCoV-Sample 111	I	European Nucleotide Archive	HF954928.1
FCoV-Sample 125	I	European Nucleotide Archive	HF954929.1
FCoV-Sample 126	I	European Nucleotide Archive	HF954930.1
FCoV-Sample 128	I	European Nucleotide Archive	HF954931.1
FCoV-Sample 129	I	European Nucleotide Archive	HF954932.1
FCoV-Sample 131	I	European Nucleotide Archive	HF954933.1
FCoV-Sample 07 129308-1	I	European Nucleotide Archive	HF954957.1
FCoV-Sample 08 153990-1	I	European Nucleotide Archive	HF954958.1
FCoV-Sample D04 93-1	I	European Nucleotide Archive	HF954968.1
FCoV-Sample D05 77-1	I	European Nucleotide Archive	HF954955.1
FCoV-Sample D06 244-1	I	European Nucleotide Archive	HF954953.1
FCoV-Sample D06 327-1	I	European Nucleotide Archive	HF954951.1
FCoV-Sample N05 48-1	I	European Nucleotide Archive	HF954962.1
FCoV-WSU-79-1683	II	NCBI GenBank	AFH58021.1
FCoV-WSU-79-1146	II	NCBI GenBank	AAY32596.1
FCoV-DF-2	II	NCBI GenBank	AFH55111.1
FCoV-NTU156/P/2007	II	NCBI GenBank	ACS44218.1
FCoV-M91-267	II	NCBI GenBank	BAN67900.1
FCoV-KUK-H/L	II	NCBI GenBank	BAN67909.1
FCoV-Tokyo/cat/130627	II	NCBI GenBank	BAP19067.1
